# Analytical Thermal Modeling of Powder Bed Metal Additive Manufacturing Considering Powder Size Variation and Packing

**DOI:** 10.3390/ma13081988

**Published:** 2020-04-24

**Authors:** Jinqiang Ning, Wenjia Wang, Xuan Ning, Daniel E. Sievers, Hamid Garmestani, Steven Y. Liang

**Affiliations:** 1 George W. Woodruff School of Mechanical Engineering, Georgia Institute of Technology, 801 Ferst Drive, Atlanta, GA 30332, USA; wenjia@gatech.edu; 2 School of Mathematics, Georgia Institute of Technology, 686 Cherry St NW, Atlanta, GA 30332, USA; xning32@gatech.edu; 3 The Boeing Company, 499 Boeing Boulevard, Huntsville, AL 35824, USA; Elliott.sievers@gmail.com; 4 School of Materials Science and Engineering, Georgia Institute of Technology, 771 Ferst Drive NW, Atlanta, GA 30332, USA; hamid.garmestani@mse.gatech.edu

**Keywords:** PBMAM, analytical thermal modeling, powder material property calculation, powder size distribution and packing, AlSi10Mg

## Abstract

This work presents a computationally efficient predictive model based on solid heat transfer for temperature profiles in powder bed metal additive manufacturing (PBMAM) considering the heat transfer boundary condition and powder material properties. A point moving heat source model is used for the three-dimensional temperature prediction in an absolute coordinate. The heat loss from convection and radiation is calculated using a heat sink solution with a mathematically discretized boundary considering non-uniform temperatures and heat loss at the boundary. Powder material properties are calculated considering powder size statistical distribution and powder packing. The spatially uniform and temperature-independent material properties are employed in the temperature prediction. The presented model was tested in PBMAM of AlSi10Mg under different process conditions. The calculations of material properties are needed for AlSi10Mg because of the significant difference in thermal conductivity between powder form and solid bulk form. Close agreement is observed upon experimental validation on the molten pool dimensions.

## 1. Introduction

Powder bed metal additive manufacturing (PBMAM), also referred to as powder bed fusion (PBF), can produce geometrically complex parts in a single unit or small batch with effective cost [1]. High-density laser power is employed in PBMAM to fully melt and fuse metal powders to build parts in a layer-by-layer manner [2]. Due to the large thermal gradient from repeatedly rapid heat and solidification during the process, defects such as porosity and cracks [3,4], undesired residual stress [5,6], fatigue [7,8], and part distortion [9,10] are frequently observed in the produced parts. Therefore, temperature monitoring and control are needed. Different approaches were developed for such purpose, which can be broadly classified into three categories: experimental measurement, numerical modeling, and analytical modeling.

Experimental techniques were employed for the temperature measurement in metal additive manufacturing (MAM) processes. In-situ measurements are inconvenient and difficult due to the restricted accessibility under elevated temperature levels. Contact methods such as the embedded thermocouple and non-contact methods such as infrared pyrometer and infrared camera are used for the in-situ temperature measurements [11,12]. The former only measures temperature at far-field, typically inside the substrate [13]. The latter only measures temperature on the exposed surfaces [14]. Post-process measurements were developed based on the solidification microstructure, which required extensive experimental works in sample preparation using cutting, polishing, and etching [15].

To overcome the limitations in experimental techniques, finite element analysis (FEA)-based numerical models and physics-based analytical models were developed for the temperature prediction. Numerical models have made considerable progress in the prediction of MAM processes. Roberts et al. developed a numerical model for the thermal prediction in PBMAM using element birth and death technique [16]. Fu et al. developed another numerical model for the thermal prediction in PBMAM experimental solid bulk material properties and experimental powder material properties [17]. Criales et al. performed a sensitivity analysis of material properties and process parameters in the thermal prediction of PBMAM [18]. The numerical modeling has been reported and discussed in the review literature [19,20]. A detailed discussion of numerical models is out of the scope of this work. The developed numerical models rely on the iterative calculation and thus have high computational cost, which is the major limitation.

Analytical models were for temperature prediction in PBMAM without resorting to the FEA or iteration-based simulations, which significantly reduced the expensive computational cost. The high computational efficiency allows the temperature prediction for a large-scale part and process-parameter planning through inverse analysis. Different temperature models have been developed based on various heat sources, namely, point moving heat source, semi-ellipsoidal moving heat source, uniform moving heat source, and line moving heat source, as originally proposed by Carslaw and Jaeger [21], and Rosenthal [22]. Van Elsen et al. summarized and compared the developed moving heat source solutions [23]. Stump et al. implemented an analytical solution based on volumetric Gaussian heat flux with Forward Time Stepping approach for temperature prediction in multi-track scans [24]. Forslund et al. implemented an analytical solution based on Gaussian heat flux with time integration for the temperature prediction in multi-track scans [25]. The workpiece was assumed as a semi-infinite medium with isotropic and homogeneous material. A moving coordinate with an origin at the moving heat source location was employed in the developed temperature models. The employed moving coordinate prevents the consideration of heat transfer boundary conditions in the temperature prediction. Tan et al. and Ning et al. further developed the analytical model for temperature prediction in an absolute coordinate [26,27]. The heat loss due to convection and radiation at the part boundary was neglected. Semi-analytical models were developed to consider heat transfer boundary conditions. Peyre et al. proposed a semi-analytical model for temperature prediction in direct metal deposition (DMD), in which the deposition geometry and temperature distribution were calculated by analytical model and numerical model, respectively [28]. Yang et al. proposed another semi-analytical model for temperature prediction in SLM, in which the temperature change due to the heat input from moving laser and the heat loss at boundary were calculated by an analytical and numerical model, respectively [29]. A coarse mesh grid was used in the numerical models to avoid high computational cost but limited the prediction accuracy and predictive capability for large-scale parts. Ning et al. further developed the temperature solution with consideration of boundary heat loss by heat sink solution at a steady [30,31] and transient state [32,33]. However, solid bulk material properties are commonly used in the developed solution, which resulted in unoptimized prediction accuracy.

**Figure 1:**
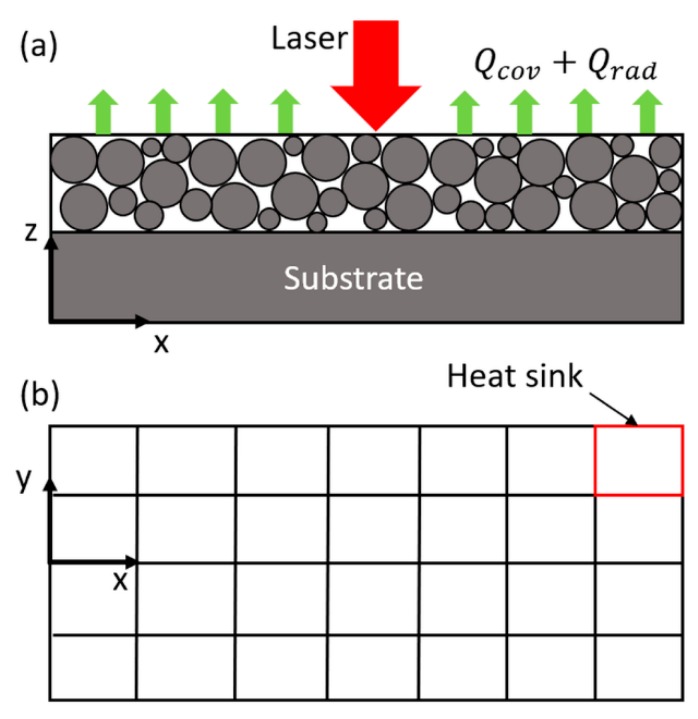
(**a**) Schematic drawing of the heat transfer mechanism in PBMAM. The red arrow represents heat input from the laser moving along positive x-direction. The green arrows represent the heat loss due to convection and radiation from the upper boundary of the powder layer. (**b**) The upper boundary of the powder layer is mathematically discretized into multiple sections (red box) due to the non-uniform temperatures and non-uniform heat loss at the boundary [34].

This work presents a physics-based analytical model to predict the three-dimensional temperature distribution in the single-track scans of PBMAM of AlSi10Mg considering the heat transfer boundary conditions and powder bed material properties. The presented model does not involve any FEA or iterative calculation-based simulations. The temperature changes due to heat input from the moving laser and heat loss from boundary heat transfer are calculated by moving heat source solution and heat sink solution, respectively. The top boundary is mathematically discretized into multiple heat sinks considering non-uniform temperature distribution and non-uniform heat loss, as illustrated in Figure 1. The final temperature solution is constructed by considering the heat input and heat loss. Powder material properties are used in the temperature prediction, which are calculated with consideration of the powder size statistical distribution and powder packing. Powder materials properties are needed for AlSi10Mg because of its significant difference in material thermal properties between powder form and solid bulk form.

## 2. Methodology

This work presents a physics-based analytical model for temperature profiles in PBMAM without resorting to any iterative-calculation-based simulations. The analytical temperature solution is constructed from the point moving heat source solution and multiple heat sink solutions using calculated powder material properties considering powder size distribution and powder packing. The point moving heat source solution, the heat sink solution, and the calculation of powder material properties are discussed in detail in the following.

The point moving heat source solution, as originally by Carslaw and Jaeger [21], is employed to calculate the temperature rise due to the heat input from a moving laser heat source. It is developed based on the energy balance equation for solid materials expressed as

(1) $$\frac{\partial \rho u}{\partial t}+\frac{\partial \rho hV}{\partial x}=\nabla \cdot \left(k\nabla T\right)+\dot{q}$$

where *u* is internal energy, *h* is enthalpy, *ρ* is density, *V* is heat source moving speed, *t* is time, *x* is distance, *k* is thermal conductivity, *T* is temperature, and $\dot{q}$ is a volumetric heat source.

A heat conduction equation is derived from the energy balance equation with *V* = 0; $du=C_{p}dT$ as

(2) $$\frac{\partial^{2}T}{\partial x^{2}}+\frac{\partial^{2}T}{\partial y^{2}}+\frac{\partial^{2}T}{\partial z^{2}}=\frac{1}{\kappa }\frac{\partial T}{\partial t}+\dot{q}$$

where *x*, *y*, *z* are directions in a Cartesian coordinate, κ is thermal diffusivity ($\kappa =k/\rho_{p}C_{p}$).

The point moving heat source solution at the current time with a time range from 0 to *t* is derived from the heat conduction equation as

(3) $$\theta_{L}\left(x,\;y,\;z,\;t\right)=\frac{P\eta }{2Rk_{p}\pi^{\frac{3}{2}}}exp\left(\frac{Vx}{2\kappa_{p}}\right)\int_{\frac{R}{2\sqrt{\kappa t}}}^{\infty }exp\left[-\xi^{2}-\left(\frac{V^{2}R^{2}}{16\kappa_{p}^{2}\xi^{2}}\right)\right]d\xi$$

where $\theta_{L}$ is the temperature rise at the location (*x*, *y*, *z*) and time (*t*). η is laser absorptivity, *R* is the distance from the heat source ($R^{2}=x^{2}+y^{2}+z^{2}$), ξ is a time-related integrable variable introduced for concise expression. The laser heat source profile is not considered in the solution. 

The heat sink solution is derived from the heat source solution with an equivalent power for heat loss and zero moving velocity. The heat sink solution calculates the temperature drop due to convection and radiation from the stationary top boundary.

(4) $$Q_{conv}=Ah\left(T_{s}-T_{0}\right)$$

(5) $$Q_{rad}=A\varepsilon \sigma (T_{s}^{4}-T_{0}^{4})$$

where A is the area of the heat sink, h is the heat transfer coefficient for convection, ε is radiation emissivity, σ is Stefan-Boltzmann constant, $T_{s}$ is the temperature at the heat sink, which can be estimated using the heat source solution. $T_{0}$ is room temperature. The subscripts *conv* and *rad* denote convection and radiation, respectively.

The heat sink solution represents the boundary heat loss from the given surface area due to convection and radiation. It is expressed as

(6) $$\theta_{s}\left(x,\;y,\;z,\;t\right)=\frac{A\left[h\left(T_{s}-T_{0}\right)+\varepsilon \sigma (T_{s}^{4}-T_{0}^{4})\right]}{2Rk_{p}\pi^{\frac{3}{2}}}\int_{\frac{R}{2\sqrt{\kappa t}}}^{\infty }exp\left(-\xi^{2}\right)d\xi$$

where $\theta_{s}$ is the temperature drop at the location (*x*, *y*, *z*) and time (*t*). The part boundary is mathematically discretized into multiple sections (multiple heat sinks) considering the non-uniform temperature distribution at the part boundary, which causes the non-uniform heat loss at the part boundary.

The final temperature solution is constructed from the superposition of the point moving heat source solution and multiple heat sink solutions as

(7) $$\theta \left(x,\;y,\;z,\;t\right)=\theta_{L}\left(x,\;y,\;z,\;t\right)-\overset{n}{\underset{1}{\sum }}\theta_{s}\left(x,\;y,\;z,\;t\right)$$

where *n* is the number of heat sinks on the part boundary. The sum of multiple heat sink solutions represents the physical quantity of total heat loss at the part boundary. The presented temperature solution has the following limitations: (1) the laser heat source is assumed as a point heat source without consideration of heat source profile. (2) The liquid phase heat transfer and evaporation of the materials are not considered. 

**Figure 2:**
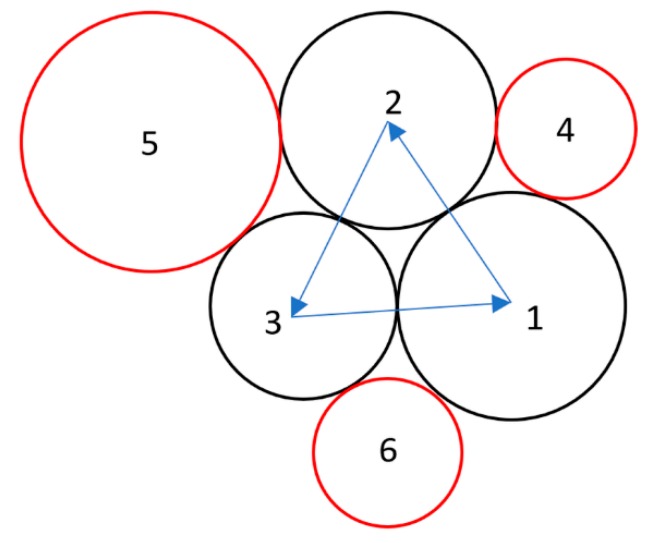
Powder bed packing with advancing front approach; 1–6 denote powders packed in sequence.

The spatially uniform and temperature-independent powder material properties are calculated and used in the temperature calculation. The powder size statistical distribution and powder packing are considered using advancing front approach within a 2D domain, as suggested by Feng et al. [35]. The different size powders are generated following a statistical powder size distribution curve and fill the 2D geometry domain without overlapping until a local maximum packing is achieved. The first three powders are mutually tangential as the first front. The additional powders are added to the initial front individually following clockwise or counterclockwise direction as shown in Figure 2. The following assumptions were made in the calculation: (1) the powders have a perfectly spherical shape, (2) the powder bed has a maximum packing density, (3) the powder spreading motion on the current layer is completed. The volume fraction of powder packing voids is calculated based on the image of packed powders. Other particle-packing algorithms, such as the rain model, considering particle size variation [36] and contact model considering regular structure (face-centered cubic FCC structure, body-centered cubic BCC structure, and simple cubic SC structure, and diamond structure) of equal size particles [37], have also been reported in the literature. The specific heat of powders and bulk materials has a negligible difference as reported in the literature [38]. 

The powder bed density is calculated as

(8) $$\rho_{p}=\left(1-\varepsilon \right)\rho_{s}+\varepsilon \rho_{g}$$

where $\rho_{p}$ is the powder bed density, $\rho_{s}$ is the solid bulk density, $\rho_{g}$ is the density of gas atmosphere (the density of air is 1.225 kg/m^3^), ε is the volume fraction of voids.

The powder bed thermal conductivity, also referred to as the effective thermal conductivity, which is calculated using a semi-empirical model considering the powder packing void and gas atmosphere [39].

(9) $$k_{p}=\frac{k_{s}(1-\varepsilon )}{1+\psi \frac{k_{s}}{k_{g}}}$$

(10) $$\psi =0.02\times 10^{2\left(\varepsilon -0.3\right)}$$

where $k_{p}$ is the powder bed thermal conductivity, $k_{s}$ is the bulk thermal conductivity, $k_{g}$ is the thermal conductivity of the gas atmosphere, and ψ is an exponential factor, ε is the volume fraction of voids. The thermal conductivity of the commonly used gas atmosphere in metal additive manufacturing is given in Table 1.

Different models have been reported in the literature to calculate the effective thermal conductivity of the powder bed. Gusarov et al. proposed a model to calculate the effective thermal conductivity considering equal-sized powder packing [37]. More information about the effective thermal conductivity of equally sized powder packing can be found in the review literature [41]. Moser et al. proposed a semi-empirical model to calculate the effective thermal conductivity considering particle–particle conduction and particle–gas–particle conduction [38]. Sih et al. proposed another model to calculate the effective thermal conductivity considering the conduction, convection, and radiation in the solid phase, liquid phase, and gas phase [42]. The presented model is used because it is effective, simple, and easy-to-use. 

## 3. Results and Discussion

**Figure 3:**
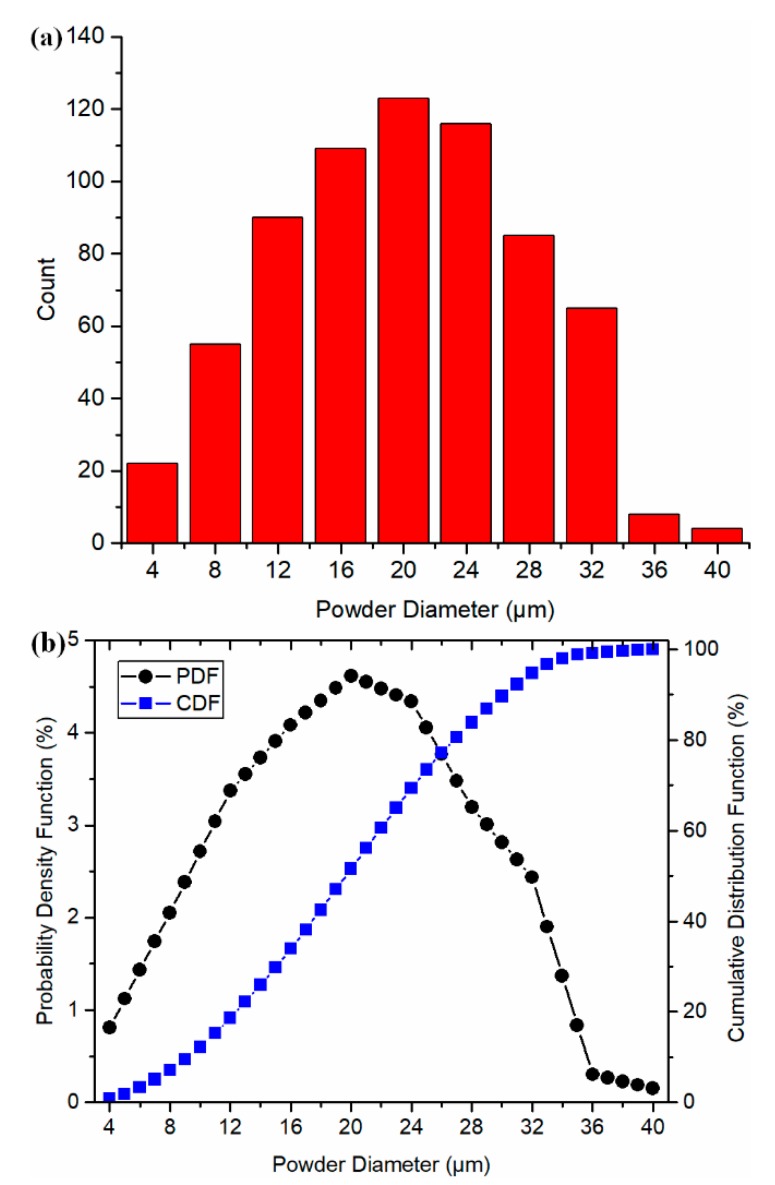
The powder size distribution of AlSi10Mg powders in this study. (**a**) Powder counts at the given powder size, and (**b**) probability density function (PDF) and cumulative distribution function (CDF) of the powder size distribution.

In this section, the presented model was employed for the temperature prediction in multiple single-track scans during PBMAM of AlSi10Mg under different process conditions. The material properties of the powder bed were used in the temperature prediction, in which the powder material properties were calculated with consideration of the powder size distribution and powder packing under the air atmosphere. The quasi-spherical shape of AlSi10Mg powders and powder size statistical distribution were reported in the literature [43], in which a Scanning Electron Microscope (SEM) was employed for the analysis. The powder size distribution curve is shown in Figure 3. The red columns represent the counts of powders at the given size. The black and blue curves represent the probability density function (PDF) and the cumulative distribution function (CDF) of the powders size distribution. The average powder size and standard deviation were extracted as 19.94 and 7.82 μm, respectively.

**Figure 4:**
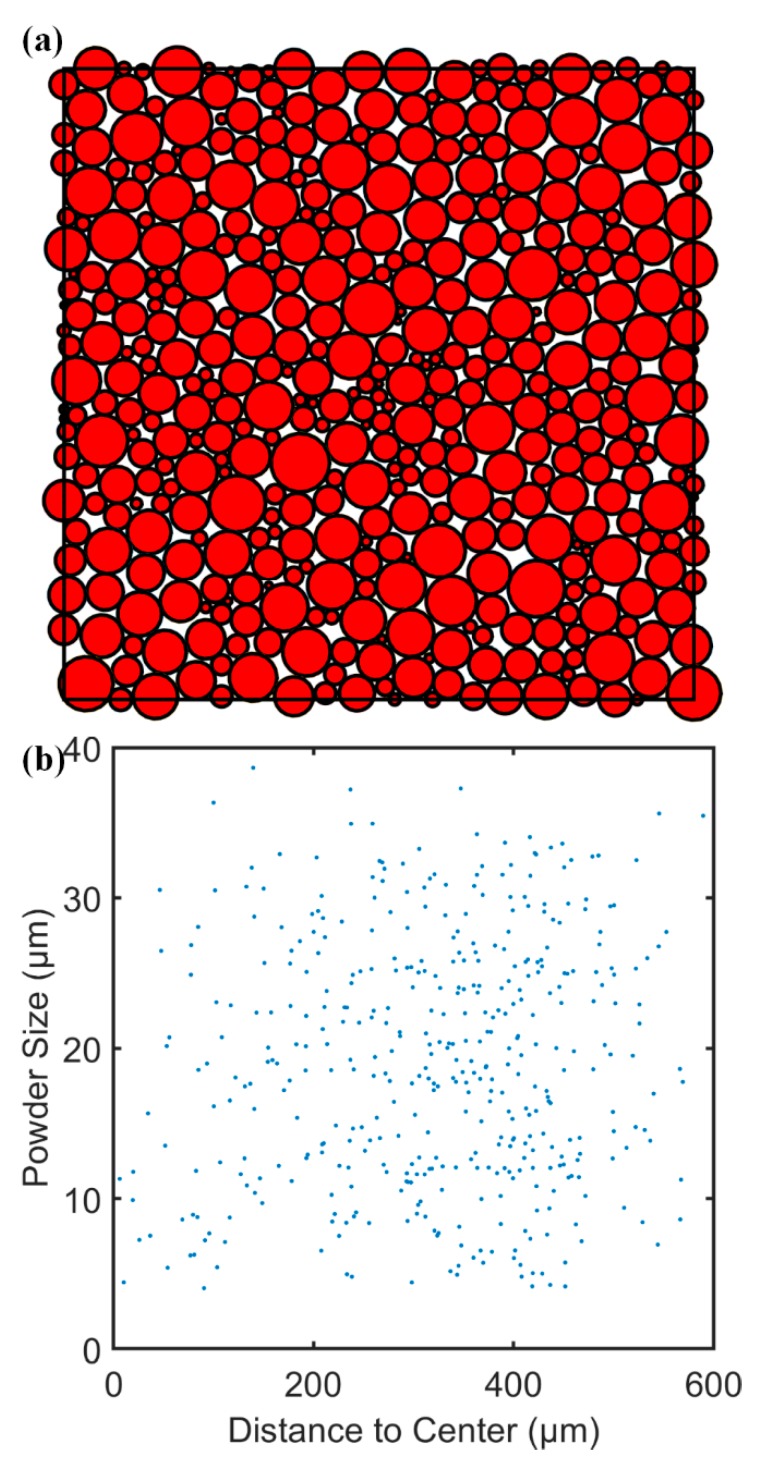
Predicted powder packing structure of ALSi10Mg in this study. (**a**) Packing structure of 500 powders, and (**b**) powder size and center location of 500 packed powders.

Spherical powders were generated based on the probability distribution curve. The powder packing structure was predicted using the advancing front approach as illustrated in Figure 4, in which the red circles and white irregular shapes represent packed powders (solid) and void, respectively. 

The influence of uncertainty in powder packing and the number of powders on void calculation was investigated with a sensitivity analysis, in which the powder packing structure was calculated three times at each level of powder numbers with the maximum value of standard deviation at 0.0021. The associated data is given in Table 2. The packing uncertainty and number of powders have a negligible influence on the calculated volume factions of void and solid. The average volume fraction of the powder packing void was determined as 0.1648.

The solid bulk materials properties of AlSi10Mg and process parameters of SLM are given in Table 3. The solid thermal conductivity and solid specific heat were given as constants at a middle temperature (581.65 K) between room temperature and the materials’ melting temperature. The heat transfer coefficient, thermophysical material properties were given as constants in the solid phase thermal analysis. The powder absorption was adopted from the literature as 0.3 [43], which were determined according to the analysis by Stacy et al. [44]. The laser absorption is related to the wavelength of the laser source, powder material properties, powder bed surface roughness, and standoff distance between laser and powder. It should be noted that this laser absorption value is only valid in this study.

**Figure 5:**
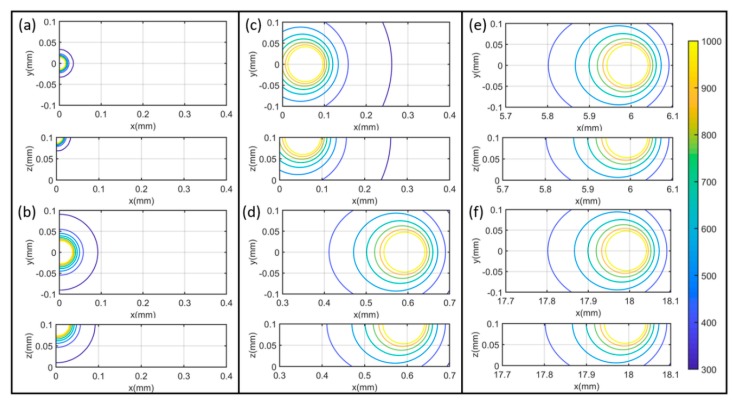
Temperature profiles during a single-track scan of PBMAM with *P* = 180 W, *V* = 600 mm/s at (**a**) 0.001 ms (**b**) 0.01 ms (**c**) 0.1 ms (**d**) 1 ms (**e**) 10 ms (**f**) 30 ms. It should be noted that the color bar is corresponding to the temperature contour value in Kelvin. The temperature plots on *x*-*y* planes are the temperature profiles at the top boundary; the temperature plots on *x*-*z* planes are the temperature profiles at the cross-sectional area along the laser scanning direction. Laser scanning direction is along positive x-direction at *y* = 0 mm.

**Figure 6:**
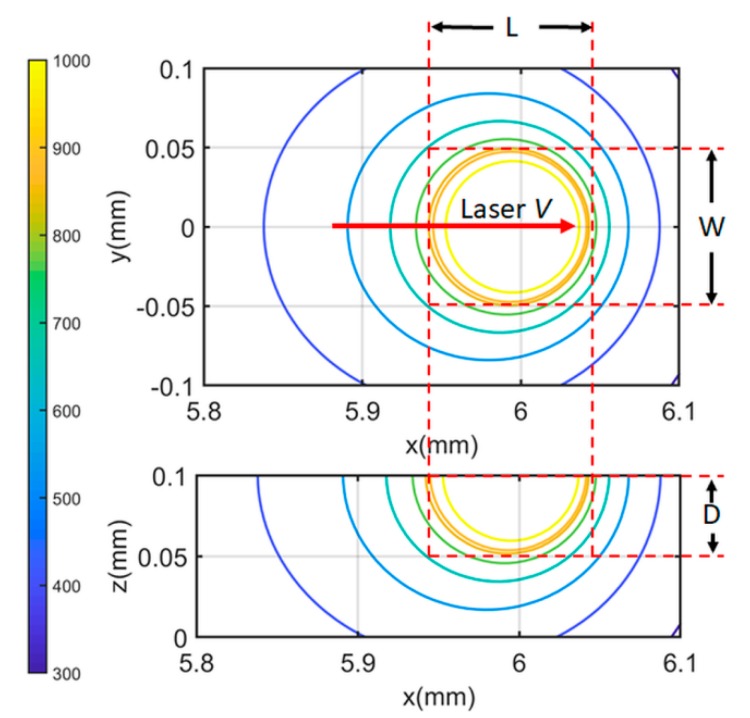
Molten pool length (L), molten pool width (W) and molten pool depth (D) calculated from the predicted temperature profile at the top boundary (*x*-*y* plane) and the predicted temperature profile at the cross-sectional area (*x*-*z* plane) at the laser scan direction along positive *x*-direction at y = 0 mm.

The heat sink area was determined as 0.001 mm^2^ with experimental calibration based on the molten pool dimensions at steady state. The temperature distribution was calculated for a region with dimensions of 0.5 by 0.2 by 0.1 mm with an increment of 5 μm in each direction in the absolute coordinate. The temperature distribution was predicted with respect to the scanning time and laser traveling distance from the beginning point. The temperature distribution at *t* = 0.0001, 0.001, 0.01, 0.1, 1, 10, 20, and 30 ms were predicted using the presented model under test 1 process condition (*P* = 180 W, *V* = 600 mm/s), as illustrated in Figure 5. The molten pool dimensions of molten pool length, molten pool width, molten pool depth were calculated by comparing to the materials’ melting temperature as illustrated in Figure 6. 

**Figure 7:**
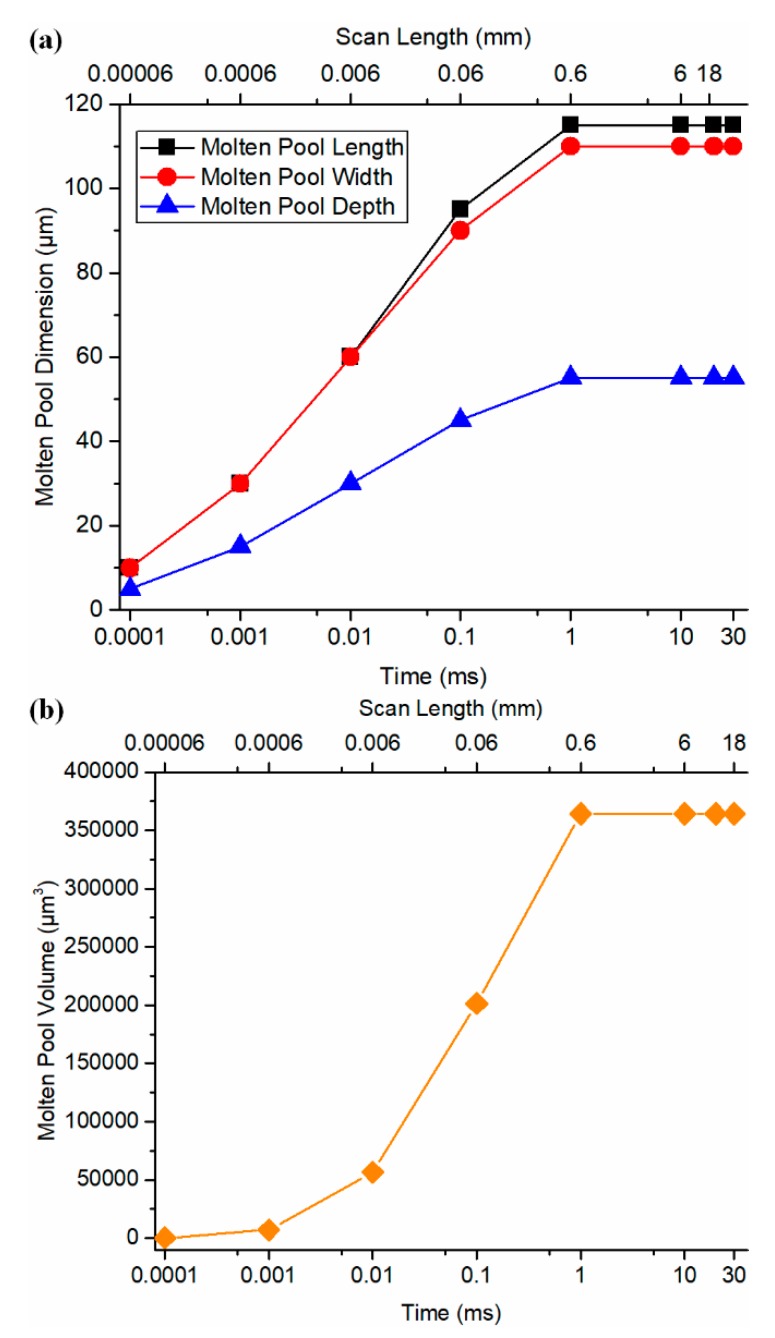
Molten pool growth and stabilization during the single-track scan of SLM of AlSi10Mg with *P* = 180W, *V* = 600 mm/s. (**a**) Molten pool length, width, and depth, and (**b**) molten pool volume.

The molten pool evolution during the single-track scan under test 1 process condition (*P* = 180 *W*, *V* = 600 mm/s) was illustrated in Figure 7. It should be noted that the cooling stage after turning the laser off is not considered in the current study. The molten pool volume was estimated by the following equation as suggested by Fu et al. [17],

(11) $$Volume=\frac{\pi DLW}{6}$$

A stabilized molten pool was observed after 1 ms (0.6 mm) under test 1 process condition. The same analyses were performed under tests 2–4 conditions. Similar trends were observed.

**Figure 8:**
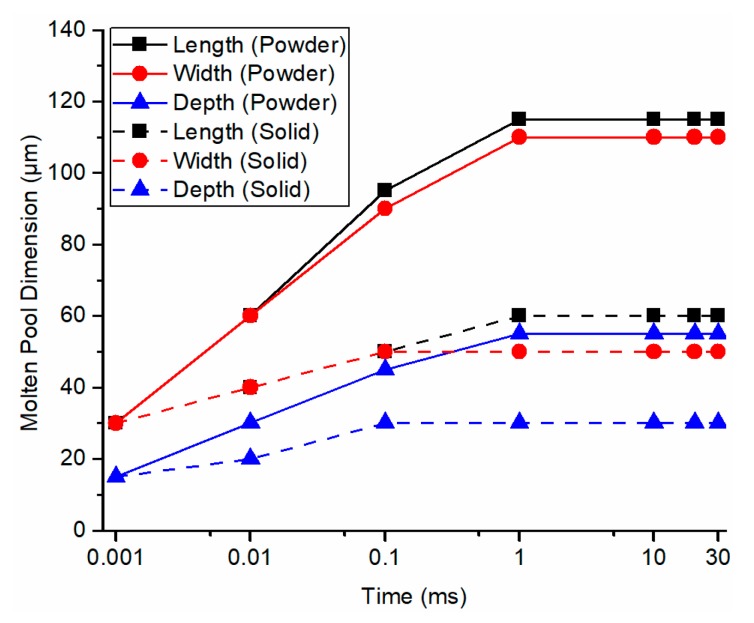
Comparison between predicted molten pool evolution using powder material properties with consideration of heat transfer boundary conditions (solid lines) and predicted molten pool evolution using solid bulk material properties without consideration of heat transfer boundary conditions (dashed lines).

**Figure 9:**
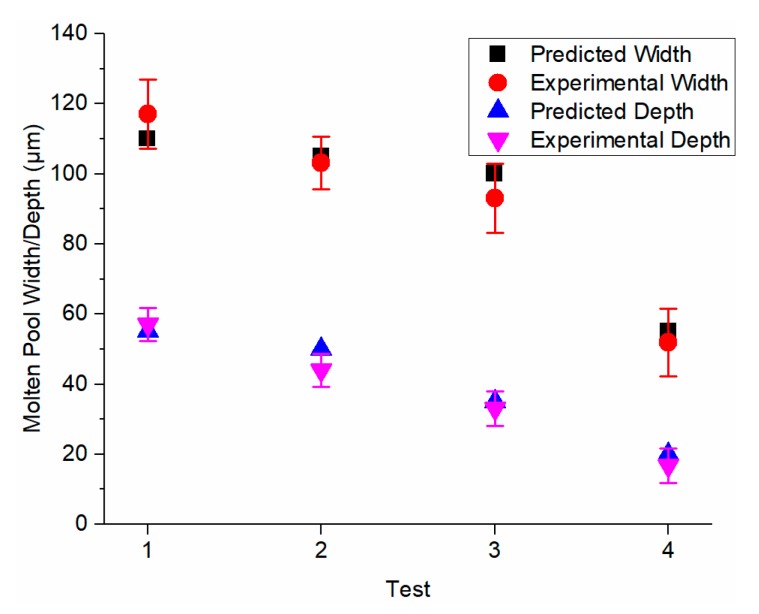
Validation of calculated molten pool dimensions to the documented experimental values based on the solidification microstructure. *P* = 180 W, *V* = 600, 800, 1000 and 1600 mm/s, respectively.

**Figure 10:**
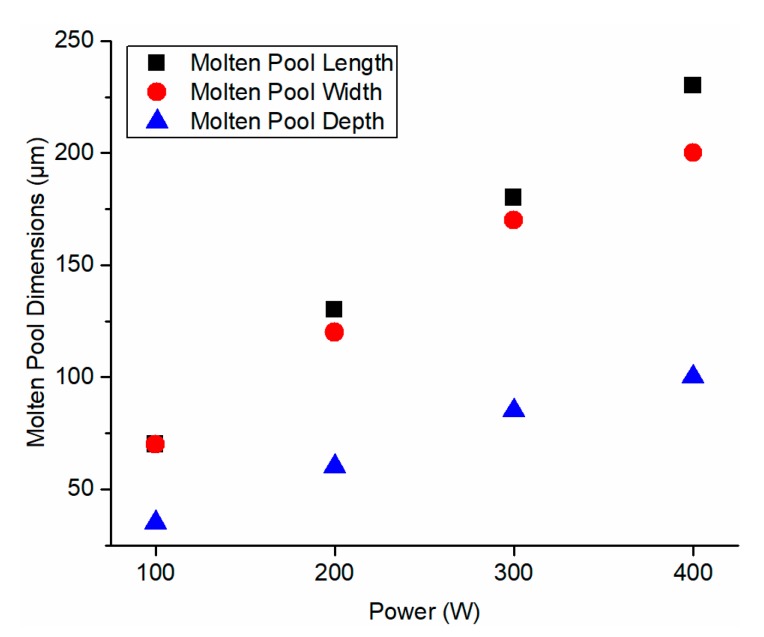
Predicted molten pool dimensions under various laser power settings. The laser scanning velocity was 600 mm/s.

For comparison, the molten pool evolution was also predicted by the developed model in the authors’ previous work [27], which used the solid bulk materials properties without considering the heat loss at the part boundary. As shown in Figure 8, the time for molten pool stabilization using the developed model in the previous work and the presented model has no pronounced difference. However, the stabilized molten pool dimensions using the presented model were significantly larger than those using the previous model because the solid thermal conductivity is significantly higher than powder thermal conductivity without introducing the voids with high thermal resistance. High thermal conductivity materials require larger laser power for the SLM process because the energy can be more easily dissipated into the surrounding area, which prevents the material melting and the formation of the molten pool. To investigate the prediction accuracy, the stabilized molten pool dimensions from the prediction using the presented model were validated to the documented experimental values in the literature [43]. Close agreements were observed between predictions and experimental values as shown in Figure 9. Other associated data including molten pool length and computational time is given in Table 4. The calculation was carried out by MATLAB program on a personal computer running at 2.8 GHz. The calculated region has dimensions of 0.4 mm in length, 0.2 mm in width, and 0.1 mm in depth with the increment of 5 μm in each direction in the absolute coordinate. The average computational time was 217.93 s. In addition, the higher the laser scanning speed, the smaller the molten pool dimensions, and vice versa. This observed trend was confirmed with the experimental measurements. Moreover, the molten pool dimensions were also predicted using the presented model with various laser power setting as illustrated in Figure 10. The higher the laser power, the larger the molten pool size, and vice versa. This observation was confirmed by instinctive trend and further prove the predictive capability of the presented model. 

The presented model has demonstrated high prediction accuracy and high computational efficiency in the temperature prediction of PBMAM. The high computational efficiency of the presented model allows the temperature prediction for a large-scale part and process-parameters planning and optimization through inverse analysis [45]. The coarse mesh resolution in the numerical model can increase computational efficiency but decrease the prediction accuracy. The presented model was developed based on the physics without resorting to FEA or any iteration-based simulations and thus a coarse calculation can further increase the computational efficiency with the prediction accuracy remained unaffected. In the future, the laser heat source profile, heat transfer in the liquid phase, the material evaporation should be considered to further improve the prediction accuracy and prediction capability. The temperature model for multi-tracks and -layer scans should be developed to further improve the usefulness of the analytical model in real applications. The temperature-dependent material properties should be considered in the temperature prediction with consideration of the heat-affected zone produced by previous scans. The part porosity [34,46], residual stress [47] and part distortion [48,49] can be further predicted from the temperature calculation.

## 4. Conclusions

This work presents a physics-based analytical model for the temperature prediction in the single-track scan of PBMAM without resorting to FEA or any iterative calculation-based simulations. The presented model does not involve FEA and thus has a promisingly short computational time. The temperature changes due to laser heat input and boundary heat loss were calculated by the point moving heat source solution and heat sink solution, respectively. The final solution was constructed from a heat source solution and heat sink solution. The powder material properties of thermal conductivity and density were calculated considering the powder packing voids, which was calculated using the advancing front approach with consideration of powder size statistical distribution and powder packing.

The temperature profiles and molten pool evolution concerning laser scanning time and laser traveling distance were predicted using the presented model under various process conditions in PBMAM of AlSi10Mg. AlSi10Mg has a significant difference in thermal conductivity between powder form and solid bulk form, and thus the accurate temperature prediction requires the calculations of powder material properties. The molten pool dimensions were calculated by comparing the predicted temperatures to the material melting temperature. The stabilized molten pool dimensions were calculated under different process conditions. Close agreements were observed upon validation to the documented experimental values. The sensitivity analyses were performed for the process parameters. A positive relationship was observed between laser power and molten pool dimensions. A negative relationship was observed between laser scanning velocity and molten pool dimensions. The computational time for each temperature prediction was recorded and reported. The advantages in prediction accuracy and computational efficiency of the presented model promise the temperature prediction for large-size part and process parameter planning and optimization through inverse analysis.

## Figures and Tables

**Table 1:** Thermal conductivity of the common gas atmosphere [38,40].

Gas	$k_{g}\;(W/\mathrm{mK})$
Helium	0.1502
Nitrogen	0.0255
Argon	0.0176
Air	0.16–0.166

**Table 2:** Volume fractions of void and solid of packed powder bed with the various number of powders.

Number of Powders	Void Volume Fraction	Solid Volume Fraction
100	0.1676	0.8324
250	0.1636	0.8364
500	0.1685	0.8315
750	0.1625	0.8375
1000	0.1620	0.8380
Average	0.1648	0.8352

**Table 3:** Materials properties of AlSi10Mg and process parameters in PBMAM [43]. The laser scan velocities for test 1–4 are 600 mm/s, 800 mm/s, 1000 mm/s, and 1600 mm/s, respectively.

Parameter	Value	Unit
Solid bulk density $\rho_{s}$	2680	kg/m^3^
Solid bulk specific heat $C_{s}$	1024	J/(kgK)
Solid bulk thermal conductivity $k_{s}$	233	W/(mK)
Heat transfer coefficient h	82	W/(m^2^K)
Radiation emissivity ε	0.4	1
Stefan-Boltzmann constant	5.67 × 10^−8^	W/(m^2^K^4^)
Room temperature $T_{0}$	293.15	K
Solidus temperature $T_{s}$	830.15	K
Liquidus temperature $T_{l}$	870.15	K
Laser power *P*	180	W
Laser scan velocity *V*	600–1600	mm/s
Powder absorption *η*	0.3	1

**Table 4:** Predicted molten pool length and computational time under test 1–4 process conditions.

Test	*L* (μm)	*t* (s)
1	115	215.79
2	110	217.49
3	100	217.30
4	70	221.13
